# Novel therapeutic approach to slow down the inflammatory cascade in acute/subacute spinal cord injury: Early immune therapy with lipopolysaccharide enhanced neuroprotective effect of combinational therapy of granulocyte colony-stimulating factor and bone-marrow mesenchymal stem cell in spinal cord injury

**DOI:** 10.3389/fncel.2022.993019

**Published:** 2022-11-23

**Authors:** Shiva Hashemizadeh, Saereh Hosseindoost, Ameneh Omidi, Hossein Aminianfar, Somayeh Ebrahimi-Barough, Jafar Ai, Babak Arjmand, Mahmoudreza Hadjighassem

**Affiliations:** ^1^Brain and Spinal Cord Injury Research Center, Neuroscience Institute, Tehran University of Medical Sciences, Tehran, Iran; ^2^Pain Research Center, Neuroscience Institute, Tehran University of Medical Sciences, Tehran, Iran; ^3^Department of Anatomical Sciences, Faculty of Medical Sciences, Tarbiat Modares University, Tehran, Iran; ^4^Institute of Biomedical Research, University of Tehran, Tehran, Iran; ^5^Department of Tissue Engineering and Applied Cell Sciences, Faculty of Advanced Technologies in Medicine, Tehran University of Medical Sciences, Tehran, Iran; ^6^Cell Therapy and Regenerative Medicine Research Center, Endocrinology and Metabolism Molecular-Cellular Sciences Institute, Tehran University of Medical Sciences, Tehran, Iran; ^7^Metabolomics and Genomics Research Center, Endocrinology and Metabolism Molecular-Cellular Sciences Institute, Tehran University of Medical Sciences, Tehran, Iran; ^8^Department of Neuroscience and Addiction Studies, School of Advanced Technologies in Medicine, Tehran University of Medical Sciences, Tehran, Iran

**Keywords:** combination therapy, inflammation, bone-marrow mesenchymal stem cell, G-CSF, LPS, spinal cord injury

## Abstract

Bone-marrow mesenchymal stem cells (BM-MSCs) have not yet proven any significant therapeutic efficacy in spinal cord injury (SCI) clinical trials, due to the hostile microenvironment of the injured spinal cord at the acute phase. This study aims to modulate the inflammatory milieu by lipopolysaccharide (LPS) and granulocyte colony-stimulating factor (G-CSF) to improve the BM-MSCs therapy. For this purpose, we determined the optimum injection time and sub-toxic dosage of LPS following a T10 contusion injury. Medium-dose LPS administration may result in a local anti-inflammatory beneficial role. This regulatory role is associated with an increase in NF-200-positive cells, significant tissue sparing, and improvement in functional recovery compared to the SCI control group. The second aim was to examine the potential ability of LPS and LPS + G-CSF combination therapy to modulate the lesion site before BM-MSC (1 × 105 cells) intra-spinal injection. Our results demonstrated combination therapy increased potency to enhance the anti-inflammatory response (IL-10 and Arg-1) and decrease inflammatory markers (TNF-α and CD86) and caspase-3 compared to BM-MSC monotherapy. Histological analysis revealed that combination groups displayed better structural remodeling than BM-MSC monotherapy. In addition, Basso–Beattie–Bresnahan (BBB) scores show an increase in motor recovery in all treatment groups. Moreover, drug therapy shows faster recovery than BM-MSC monotherapy. Our results suggest that a sub-toxic dose of LPS provides neuroprotection to SCI and can promote the beneficial effect of BM-MSC in SCI. These findings suggest that a combination of LPS or LPS + G-CSF prior BM-MSC transplantation is a promising approach for optimizing BM-MSC-based strategies to treat SCI. However, because of the lack of some methodological limitations to examine the survival rate and ultimate fate of transplanted BM-MSCs followed by LPS administration in this study, further research needs to be done in this area. The presence of only one-time point for evaluating the inflammatory response (1 week) after SCI can be considered as one of the limitations of this study. We believed that the inclusion of additional time points would provide more information about the effect of our combination therapy on the microglia/macrophage polarization dynamic at the injured spinal cord.

## Graphical Abstract

Schematic illustration of beneficial effect of combination therapy of LPS and G-CSF before BM-MSC transplantation in the spinal cord injury model. 
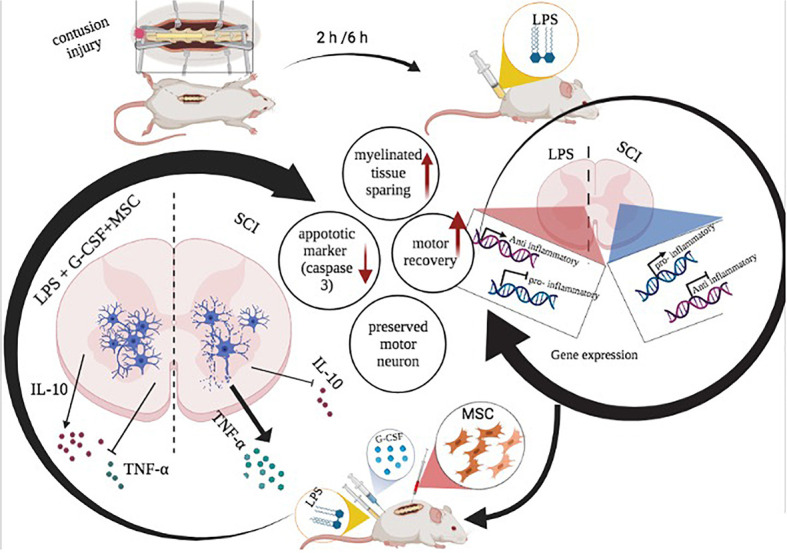

## Introduction

Traumatic spinal cord injury (SCI) is defined as an unpredictable mechanical injury to the spinal cord. Depending on the level and severity of the trauma, it could cause temporary or permanent changes in normal spinal functions, including the motor, sensory, and autonomous control (Ahuja et al., 2017). Moreover, another medical complication after SCI is also a critical issue to make the patients suffer. Until now, there is no cure to reverse damage to the spinal cord. Prevention strategies for SCI are a global mapping project. Despite numerous efforts undertaken by the International Spinal Cord Society (ISCoS), SCI is still prevalent (Bickenbach et al., 2013), occurring among individuals because of unpredictable events such as falls, road traffic crashes, and sports injuries (Devivo, 2012).

The primary lesion to the spinal cord triggers the complex multi-cascades of secondary mechanisms. It continues for months to induce significant neuropathological changes (Anjum et al., 2020). Delayed and progressive secondary mechanisms are mainly characterized by complex cellular and molecular events such as oxidative stress, excitotoxicity, and inflammatory response (Rowland et al., 2008; Oyinbo, 2011). These biochemical events create a “microenvironment imbalance” in the injured spinal cord and account for the greater portion of disability and loss of neurological function (Fan et al., 2018).

In the clinical setting, early management of SCI focuses on stabilizing patients with decompressive surgical intervention and routine use of high-dose methylprednisolone as an immunosuppressive pharmacological agent. But there is considerable uncertainty regarding the potential impact of steroid administration due to serious side effects such as infection and pneumonia (Hachem et al., 2017; Karsy and Hawryluk, 2019). Therefore, preclinical and clinical investigations over the past decades have focused to develop new therapeutic approaches in the management of SCI, including neuroprotective and regenerative strategies, cell-based therapies (Badner et al., 2017; Ahuja et al., 2020; Huang et al., 2021), and neuromodulation approaches through electrical stimulation (James et al., 2018; Yang et al., 2020).

Most new therapeutic approaches target a single mechanism of secondary injury to provide enhanced regeneration. It seems that monotherapeutic attempts failed to translate in the clinic and exert their therapeutic effects. Therefore, a multimodal approach should be considered by either a combination drug or cell-based therapy or the use of a candidate’s drug with multimodal properties, including but not limited to granulocyte colony-stimulating factor (G-CSF) (Griffin and Bradke, 2020).

Neural stem cells and mesenchymal stem cells are the most frequently used stem cell type in experimental SCI studies, indicating preliminary steps for the future clinical translation of this strategy. Over the past decades, several preclinical studies have demonstrated that MSCs as a promising source due to their feasibility and immune-privileged properties and lack of tumor-initiating potential. Therefore, potentially apply in an allogenic setting without eliciting an immunological rejection. Neuroprotective and restorative effects of MSCs are based on the bystander effect through the release of trophic factors and cytokines rather than the cell replacement when transplantation after injury to promote regeneration after SCI.

Following spinal cord trauma, a robust inflammatory response was observed. The first wave of inflammation is associated with the pro-inflammatory macrophage (M1) and reaches the peak at 7 days post-sci. The second wave corresponds to the anti-inflammatory macrophage (M2) and is maintained at least until 180 days after SCI (Milich et al., 2019). Evidence has demonstrated that the pro-inflammatory microenvironment of the injured spinal cord at the acute phase has a prominent negative impact on transplanted cells and limited therapeutic effects of BM-MSCs after transplantation (Beck et al., 2010). Research showed that early management of SCI is necessary to improve neurological recovery after SCI (Varma et al., 2013). To enhance the potential of transplanted MSCs, it is substantially important to modulate the microenvironment at the acute phase by altering inflammatory reactions. We hypothesized that the modification of the severe inflammatory processes in the microenvironment of the spinal cord by LPS combined with G-CSF treatment before MSC transplantation may enhance the therapeutic effect of cell therapy.

Lipopolysaccharide (LPS) is the main component of the outer membrane of Gram-negative bacteria that acts as an exogenous ligand of Toll-like receptor 4 (TLR-4), neurons, and resident glial cells, such as microglia, oligodendrocytes, and infiltrating macrophages express (TLR-4) (Heiman et al., 2014). More recent reports have found that TLR-4-deficient mice show poor functional motor recovery relative to wild-type SCI models (Church et al., 2016). This suggests that TLR-4 is a promising target for modulating the inflammatory response to facilitate spinal cord regeneration.

Granulocyte colony-stimulating factor is a 19 kDa glycoprotein as a growth factor that has been approved to mobilize bone marrow hematopoietic progenitor cells for transplantation. The G-CSF also promotes functional recovery by neuroprotection and modulates inflammation by switching the phenotype of microglia toward the anti-inflammatory phenotype in the injured spinal cord (Guo et al., 2013). Alternatively, the pleiotropic features of these drugs are suitable candidates for a multimodal approach against SCI.

We designed an investigation to create a permissive environment for cell transplantation at the acute phase. Therefore, in the present *in vivo* study, we examined possible combination therapeutic approaches for SCI with the use of drug administration to address early bone-marrow mesenchymal stem cells (BM-MSCs) transplantation. The first aim of the present study was to determine the optimal initial time and dose of systemic LPS alone to modulate local inflammation following SCI. The second aim of our study was to apply adjunct treatment of LPS and G-CSF with BM-MSC to enhance the beneficial effects of early transplanted stem cells to treat the clinically relevant rat spinal cord contusion injury. We investigated the inflammatory cytokine, histological outcomes, and functional recovery. Here, we report that while stand-alone therapies of BM-MSC transplantation treatment demonstrated a moderate degree of efficacy, pre-transplantation LPS injection provided synergistic effects as evidenced by high reduction in moto-neuronal cell loss, much more robust reduction in neuro-inflammation, and early improvement in locomotor recovery in a contusion model of SCI compared to stand BM-MSC mono therapies.

## Materials and methods

### Rat model of contusion spinal cord injury and postoperative care

The experiment was performed on an adult male Wistar rat (weighing 260 ± 10 g at the time of surgery) provided by the faculty of pharmacy, Tehran University of Medical Sciences. Animals were housed under standard laboratory conditions with an alternating 12-h light/dark cycle (lights on at 7 a.m.) at a constant temperature (21 ± 2°C) and free access to water and food. In this study, we designed a customized and cost-effective weight drop instrument based on the weight drop Allen model to induce contusive SCI (Hashemizadeh et al., 2022). Under sterile surgical conditions, the rats were anesthetized using a mixture of ketamine (80 mg/kg) and xylazine (10 mg/kg). After the removal of hair, a midline dorsal incision was done on the rats followed by laminectomy at T10–T12 vertebrates. Then, the rat was placed on an impactor device, and the vertebral column was fixed with two forceps rostral and caudal to the laminectomy site. Finally, the metal rod was released and crushed the spinal cord. Next, the injured muscles and skin closed with a suture. Postoperatively, animals were administrated subcutaneously 1 ml of 0.9% saline, and also rats were given 5 mg/kg of gentamicin intraperitoneally once a day until 5 days following the operation to prevent post-surgical infection. In all rats, the bladder was manually expressed twice a day until spontaneous bladder function returned.

### Experimental animals groups

#### Experiment I: Dose–response and therapeutic time window of lipopolysaccharide against spinal cord injury

**FIGURE 1:**
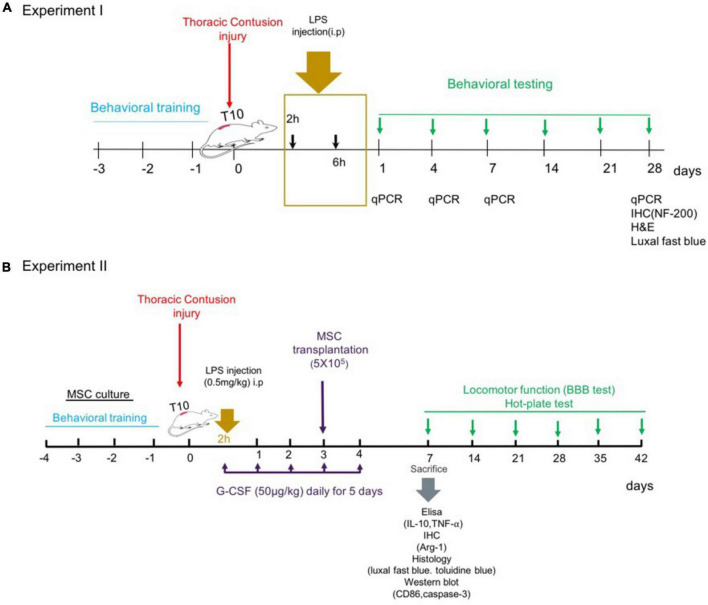
Experiments timeline. **(A)** Experiment 1: dose–response of LPS treatment. **(B)** Experiment 2: combination therapy strategy.

Rats were randomly assigned into an injury group and four treatment groups with LPS (derived from *Escherichia coli* serotype 055: B5, Sigma-Aldrich). LPS diluted in sterile pyrogen-free distilled water immediately before use at the desired concentration. Each dose of LPS was administered in equal volumes. Rats received a single i.p. injection of LPS solution as follows (Figure 1A):

The rats were randomly divided into five groups (*n* = 15 for each group, a total of 75 rats):

Group 1 – spinal cord injury group (SCI).

Group 2 – spinal cord injury group treated with a single injection of a low dose, 0.2 mg/kg of LPS, started (2 h: immediate phase) after SCI induction (SCI + low dose LPS-2 h).

Group 3 – spinal cord injury group treated with a single injection of a low dose, 0.2 mg/kg of LPS, started early (6 h: early) after SCI induction (SCI + low dose LPS-6 h).

Group 4 – spinal cord injury group treated with a single injection of a medium dose, 0.5 mg/kg of LPS, started (2 h: immediate phase) post-SCI (SCI + medium-dose LPS-2 h).

Group 5 – spinal cord injury group treated with a single injection of a medium dose, 0.5 mg/kg of LPS, started (6 h: early phase) post-SCI (SCI + medium-dose LPS-6 h).

Every treatment group was additionally divided into four time points of analysis; i.e., day 1, day 4, day 7, and 4 weeks. Three rats from each group were sacrificed for mRNA-level expression by RT-PCR on each test day. And at the fourth week, other three rats were used for histological analyses. And *n* = 6 animals per group were used for the functional test.

In group 1, two rats died of infection, in the first and second weeks. One rat from group 2 and one from group 3 died because of a refractory urinary infection on the 8 and 11 postoperative days, respectively. One rat in group 4 and one from group 5 died early in the experiment due to bladder rupture during the early post-operative care regimen.

#### Experiment II: Effects of the early administration of lipopolysaccharide combined with granulocyte colony-stimulating factor treatment and bone-marrow mesenchymal stem cell transplantation on morphological change and functional recovery

Based on the experiment of the dose–response and therapeutic window for LPS against experimental SCI, we first selected rats treated with the dose where the best behavioral and expression level was obtained. Then, we investigated the effect of combination therapy with G-CSF and BM-MSC transplantation on motoneuron survival and cytokine release and the Basso–Beattie–Bresnahan (BBB) score after SCI. G-CSF (50 μg/kg/day, Filgrastim, Neupogen) was administered 2 h post-SCI subcutaneously for 5 consecutive days. Animals received bone marrow mesenchymal stem cells (BMSC) transplants 3 days following the injury. The animals were allowed to survive for 1 week after SCI to examine inflammatory and apoptosis following the treatment approach, in addition, the animals were allowed to survive for 5 weeks after transplantation to evaluate functional recovery (Figure 1B).

A total of 62 Animals were randomly assigned to eight groups as follows: (*n* = 8 rats per group).

1) SCI control group: animals received SCI.
2) LPS group: receive a single medium dose of LPS-2 h post-SCI.
3) LPS + G-CSF group: receive a single medium dose of LPS-2 h post-SCI and daily injections of 50 μg/kg of G-CSF for 5 days.
4) SCI + BM-MSC transplantation group: received BM-MSC intra-spinal 3 days after SCI.
5) SCI + LPS + BM-MSC transplantation group: received a single medium dose of LPS-2 h post-SCI and intra-spinal BM-MSC for 3 days after SCI.
6) SCI + LPS + G-CSF + BM-MSC transplantation group: received a single medium dose of LPS-2 h post-SCI and daily injections of 50 μg/kg of G-CSF for 5 days and intra-spinal BM-MSC 3 days after.
7) SCI + vehicle group: received intra-spinal PBS at 3 days post-SCI.
8) Sham group: (*n* = 6).

In group 1, one rat died of infection, in the second week. One rat from group 2 and two rats from group 3 died because of a refractory urinary infection on the second week postoperative day. One rat in group 4 and one from group 5 died in the second week. Two rats in group 6 died because of refractory urinary infection on the second-week and fifth-week postoperative day. Four rats of group 7 were sacrificed due to sickness behavior or urinary infection.

#### Behavioral assessment

##### Hind limb locomotor score

Hind-limb motor function of all rats was evaluated using the open-field BBB rating scale as described previously by Basso et al. (1995). Motor behavior of animals was assessed 1 day before the injury and hind limb motor function was assessed on day 1, day 4, and then once weekly for the next 4 or 6 weeks. The locomotor function of each rat was evaluated by two observers who were blinded to the treatment group for 4 min.

##### Test for neuropathic pain

The hotplate test (borj sanat, Tehran) was used to evaluate thermal nociception. During the tests, the rats were placed on a metal surface having a constant temperature of 52°C ± 0.5°C. The response latency which is the time taken to observe a nociceptive behavior from the plate (foot-licking, jumping, or rapidly removing paw or vocalization) was recorded. The rats were removed from the hotplate surface once a reaction was observed or after a 20-s cut-off time if no response was observed to prevent tissue damage at 52°C. Each animal was tested twice, separated by 30 min intervals between them.

### RNA isolation, a quantitative real-time polymerase chain reaction

Injured spinal cord segments of the control and treatment groups were isolated at 1, 4, 7, and 28 days after injury. The total RNA was extracted from the homogenized spinal cord tissue with TRIzol reagent (bio basic) according to the manufacturer’s instructions. The RNA concentration in each sample was measured using NanoDrop (Thermo Fisher Scientific, Waltham, MA, USA). Then, 1 μg of purified RNA was reverse-transcripted into First-strand cDNA using a cDNA Synthesis Kit (biofact, South Korea). To measure gene expression, quantitative PCR was performed using a cyber green PCR master mix with a high ROX (biofact) on a StepOnePlus Real-Tim PCR Applied Biosystem. Real-time PCR was conducted in triplicate with each RNA sample. The program was as follows, 95°C for 15 min. followed by 45 cycles of 15 s at 94, 60°C for 15 s, and 72°C for 30 s in sequence. The forward and reverse primers used for RT-qPCR were designed using the online software NCBI/Primer-BLAST listed in Table 1. GAPDH was used as an internal control. The relative expression level of the target gene was analyzed using a 2^–ΔΔCt)^ method. A primer sequence was used for qPCR.

**TABLE 1:** Primer sequences used for real-time PCR.

Accession number	Gene	Forward primer (5′ – 3′)	Reverse primer (5′ – 3′)	Product (bp)
NM-020081	CD86	ACTTCTGTGCTGTCTCTTTCTG	ACTCACAAGTCTTTCTGCTGG	101
NM-022611.1	IL-12	ATCATCAAACCGGACCCACC	CAGGAGTCAGGGTACTCCCA	89
NM-031512.2	IL-1β	GGATGATGACGACCTGCT	ACTTGTTGGCTTATGTTCTGTC	147
NM-001276711.1	NF-κB	CGACACCTCTACACATAGCAG	CTCATCTTCTCCAGCCTTCTC	143
NM-001106123.2	Mrc1 (CD206)	GATTGACCAGTTCCTTGACCT	AACACATTCCAGATTCTCCCA	197
NM-012854.2	IL-10	CTATGTTGCCTGCTCTTACTG	CCCAAGTAACCCTTAAAGTCCT	218
NM-024125.5	C/EBPβ	ATCGACTTCAGCCCCTACCT	GGCTCACGTAACCGTAGTCG	154
NM-053828.1	IL-13	CATGGTATGGAGCGTGGA	CATTCAATATCCTCTGGGTCCT	110
NM-017008.4	GAPDH	GGAGAAACCTGCCAAGTATG	AAGAATGGGAGTTGCTGTTG	131

### Primary culture of rat bone marrow mesenchymal stem cells *in vitro* and intra-spinal grafting of stem cells

Bone marrow mesenchymal stem cell primary culture was prepared as described previously (Sangeetha et al., 2017). BMSCs were isolated and cultured from the bone marrow of the femur and tibia bones collected from transgenic adult rats expressing enhanced green fluorescent protein (EGFP). Before all experiments using BMSCs transplants, cells were characterized using flow cytometry at the fourth passage to confirm phenotypically BMSCs. Cryo-freeze allogeneic MSC transplantation was performed 3 days after the contusion injury. Cells were thawed on the day of transplantation. The rats were re-anesthetized, and the injury site was re-exposed. Animals in the MSC-treated group received transplants of (5 × 105) BM-MSC in 10-μl sterile PBS into the lesion site using a Hamilton syringe. The vehicle group was injected with 10 μl PBS alone. To maximize the engraftment of all injected BMSCs into the spinal cord, the needle was kept in place for 5 min after the injection.

### Determination of spinal cord tissue TNF-α and IL-10 levels by enzyme-linked immunosorbent assay

Cytokine levels were carried out on samples from the lesioned segment at 7 days after SCI. The section of the injured spinal cord was homogenized. The homogenates were placed in RIPA buffer and centrifuged at 13,000 × *g* for 20 min at 4°C. The supernatants were gathered, aliquoted on ice, and placed in a −80°C freezer. The expression of TNF-α and IL-10 in the injured segment was measured (*n* = 3 rat/per group) using an ELISA kit, according to the manufacturer’s protocols. The concentrations of cytokines are calculated from standard curves and are expressed in pg/ml.

### Western blotting analysis

Proteins of different groups in the injured spinal cord were collected after 7 days post-SCI. Protein concentration was measured with the Bradford assay. An equal amount of protein (40 μg per well) was loaded to separate on 12.5% SDS-PAGE and transferred onto PVDF membranes. Thereafter, the membranes were blocked in 5% fat-free skimmed milk in a Tris-buffered saline solution for 1 h at room temperature. Membranes were incubated with primary antibody against CD86 (Santa Cruz Biotechnology) caspase-3 (1:2,000) (Cell Signaling Technology) and β-actin (1:2,500) (PADZA CO., Tehran, Iran) overnight at 4°C. After washing with TBST and then exposed to peroxidase-conjugated secondary antibody (1:5,000) for 1 h at room temperature. Finally, they were washed with TBST, and protein band detection was performed by the chemiluminescent substrate (Amersham Biosciences, Freiburg, Germany) (Hosseindoost et al., 2020).

### Histochemistry

#### Histological processing

Animals were anesthetized with an intraperitoneal injection of ketamine and xylazine, then transcardially perfused with 0.9% saline, followed by 4% paraformaldehyde in phosphate buffer (pH = 7.4). A 1 cm section of the spinal cord segment, centered at the injury epicenter was dissected, post-fixed with the same fixative for 1 week, and embedded in paraffin. Serial 7-μm transverse sections with a distance of a 350-μm interval between sections were made and stained with hematoxylin/eosin and toluidine blue staining (to count the number of motoneurons) and luxol fast blue staining (to identify myelinated areas and residual spared tissue in the thoracic spinal cord).

Finally, an experienced pathologist, blinded to the activities of the treatment groups, evaluated histopathological findings, including hyperemia, degeneration, and cellular infiltration, demyelination assigning a score from 0 = (Negative); 1 = (Mild); 2 = (Moderate); and 3 = (Severe).

#### Immunohistochemistry

Paraffin specimens of 7-μm thickness were stained with rabbit polyclonal antibody against neurofilament 200 kDa (1:1,000, Abcam, Canada) or mouse polyclonal antibody against Arg-1 (GenomeMe, Germany). After that, sections were treated with biotinylated goat polyclonal secondary antibody to rabbit IgG (1:200, Abcam, Canada) and then incubated with the streptavidin-horseradish peroxidase complex. Finally, tissues were stained with DAB followed by the counterstaining of hematoxylin.

### Statistic

All investigators involved in data acquisition or analysis were blind to group designation or experimental manipulation. Data were performed using PRISM7 software are presented as mean ± SEM. Differences between groups were investigated using one-way analysis of variance (ANOVA) followed by *post hoc* Tukey’s. For the evaluation of the BBB score overtime, an ANOVA was used with repeated measures. *P*-values < 0.05 were considered to indicate statistical significance.

## Results

### Dose–response and therapeutic time window of lipopolysaccharide against spinal cord injury

For determining the dose and therapeutic window of LPS treatment after SCI, a low dose (0.2 mg/kg) and medium dose (0.5 mg/kg) of LPS administered at immediate (2 h) or early (6 h) time post-SCI. The neurological scores and histological evaluation (H&E and LFB) and immunohistochemical staining (NF-200) were examined to measure the long-term effect of single LPS treatment following SCI.

#### Contusion spinal cord injury induces changes in mRNA expression levels of pro-inflammatory and anti-inflammatory markers over time

To determine the inflammatory response following contusion SCI, we evaluated the gene expression profile associated with pro- and anti-inflammatory markers in the injured spinal segment. First, we subjected rats to SCI, and the lesion site was collected on day 1 (early microglia response before the macrophages infiltration to the injury site), day 4 (early influx of macrophages from the peripheral circulation), day 7 (reaches a macrophage infiltration peak at the lesion site), and day 28 (chronic phase of injury). The mRNA expression of target genes compared with control (un-injured) spinal cord tissue.

**FIGURE 2:**
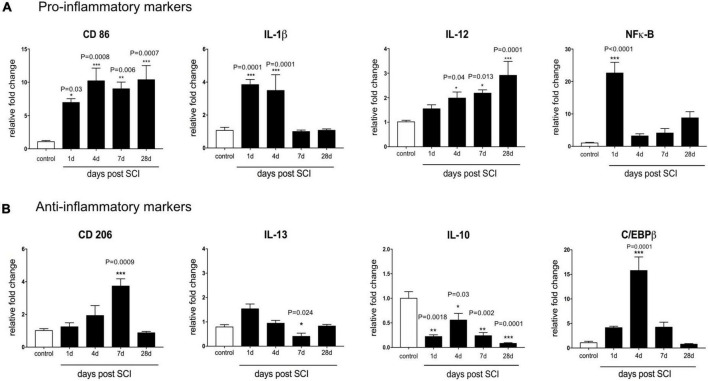
Temporal changes in the mRNA expression level of inflammatory markers after contusive spinal cord injury. Real-time PCR quantification was used to evaluate the expression level of inflammatory-associated genes in the injured spinal cord at 1, 4, 7, and 28 days post-SCI. **(A)** SCI-induced pro-inflammatory activation genes included CD86, IL-12, IL-1β, and NF-κB. **(B)** Anti-inflammatory markers included CD206, IL-10, IL-13, and C/EBPβ which were downregulated following SCI. *n* = 3 for each group. One-way ANOVA with Dunnett multiple comparison tests was used to compare gene expression levels (*P< 0.05, **P< 0.01, ***P < 0.001). Data represented as mean ± SEM fold changes of gene expression.

Figure 2A shows the mRNA levels of pro-inflammatory gene markers, including CD86, IL-12, IL-1β, and NF-κB following SCI for up to 28 days. We observed that the level of the surface marker CD86 rapidly increased after SCI from 1 to 28 days. Maximal CD86 mRNA level was observed at day 4 (*P* < 0.001) and day 28 post-SCI (*P* < 0.001) (∼10-fold versus control). Similarly, IL-12 transcript was up-regulated during 28 days after SCI and increased significantly at day 4 (*P* < 0.01), day 7 (*P* < 0.01) and maintained up to day 28 post-SCI (*P* < 0.001). Our result showed that SCI made transient up-regulation of IL-1β, which may last for up to 4 days (day 1, *P* < 0.01) but returned to the control level by 7 days after SCI. mRNA level of NF-κB evaluated as the main transcription factor to regulate other inflammatory genes. This marker rapidly increased 24 h after injury (30-fold more than normal spinal cord), but the expression level declined by 3. 2-, 4. 1-, and 8-folds, respectively, at 4, 7, and 28 days.

We next assessed the impact of contusion SCI on the expression level of an anti-inflammatory marker at the mRNA level using CD206, IL-10, IL-13, and C/EBPβ in the spinal cord tissue. Figure 2B shows that, at the transcriptional level, the injured spinal cord showed a CD206 level close to the control group between days 1 and 4. CD206 transcription raised almost fourfold (*P* < 0.001) at day 7 compared to the un-treated group. Furthermore, we observed mRNA IL-10, an anti-inflammatory cytokine was modestly decreased below the baseline level during 28 days after SCI. Also, 24 h after SCI, the expression level of IL-13 shows a non-significantly increase. However, it gradually decreased at day 7 (*P* < 0.05) and day 28 (*P* < 0.01) in the injured spinal cord. C/EBPβ as a main transcriptional regulator of M2 macrophage/microglia specific gene, up-regulated 1 week following SCI reaching the peak of nearly 11-fold at 4 days after SCI, and then reached the baseline level within 4 weeks. This result indicates that contusion SCI caused an imbalanced inflammatory milieu after SCI. It seems that the mRNA expression levels of anti-inflammatory markers reduced in favor of an increase in the mRNA expression level of pro-inflammatory markers up to 4 weeks.

#### Effects of systemic low and medium doses of lipopolysaccharide on spinal cord injury-induced mRNA changes of inflammatory marker in the lesion site

**FIGURE 3:**
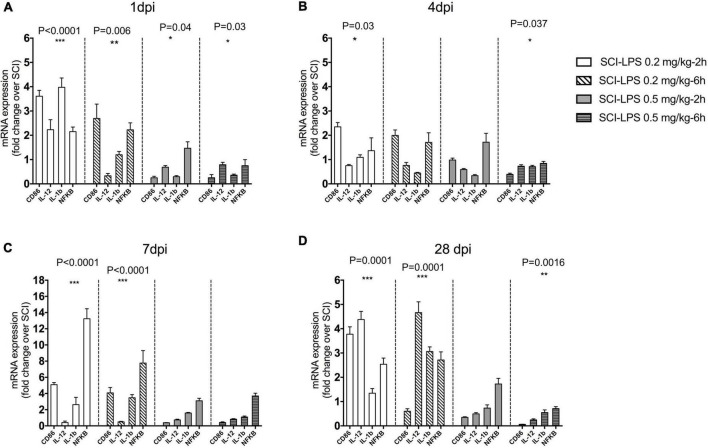
The effect of immediate (2 h) and early (6 h) administration of two sub-toxic doses of LPS on a pro-inflammatory marker after SCI. Adult male rats received the moderate contusion SCI and LPS treatment initiated at 2 or 6 h post-SCI. Real-time PCR quantification was performed to evaluate pro-inflammatory markers at 1 **(A)**, 4 **(B)** 7 **(C)**, and 28 **(D)** days after SCI. Gene expression is normalized to the same-day SCI group. Regardless of injection time, LPS 0.5 mg/kg significantly reduces mRNA level pro-inflammatory at 1, 4, and 28 dpi compared to the SCI. There was a trend to increase pro-inflammatory gene during 28 days after the SCI in low-dose LPS (0.2 mg/kg) treatment groups. Days *P < 0.05, **P < 0.01, ***P < 0.001 versus SCI (main effect of treatment), *n* = 3, mean + SEM *n* = 3 for each group.

Next, we used the inflammatory genes to determine the dose-dependent effect of early LPS treatment on the SCI inflammatory milieu. LPS was delivered 2 or 6 h after SCI. Then the injured spinal cord was isolated at 1, 4, 7, and 28 days post injury for gene analysis. There were significant main effects of treatment for pro-inflammatory gene expression at both 1 dpi [(*F*_12,86_) = 8.027, *P*< 0.0001, *n* = 3] and 4 dpi [(*F*_12,89_) = 5.407, *P*< 0.0001, *n* = 3] and 7 dpi [(*F*_12,66_) = 18.61, *P*< 0.0001] as well 28 dpi [(*F*_12,76_) = 18.36, *P*< 0.0001]. Our results indicated that during 4 weeks after SCI, inflammatory gene expression significantly increased for all low-dose LPS treatments (*P*< 0.0001, main effect versus SCI for each treatment group). In contrast at both 1 and 4 dpi, inflammatory gene expression was significantly reduced for all medium-dose LPS treatment groups compared to SCI (*P*< 0.0001, *P* < 0.05 main effect versus SCI for each treatment group respectively, *n* = 3–4) (Figure 3).

**FIGURE 4:**
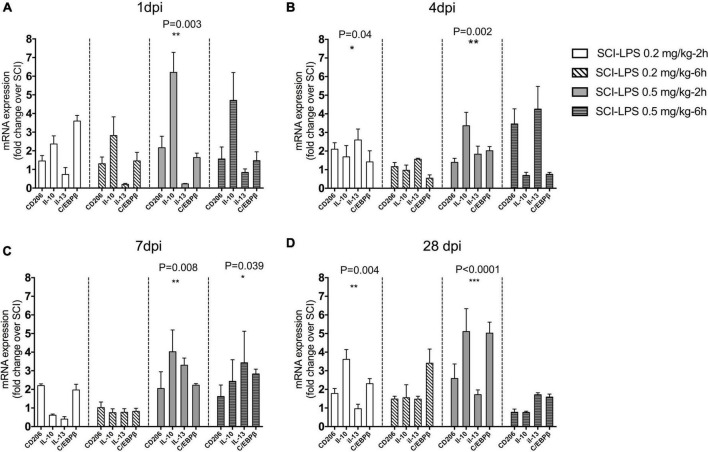
Post-spinal cord injury medium-dose LPS treatment significantly increased anti-inflammatory gene expression in the lesion center. Adult male rats received the moderate contusion SCI and LPS treatment was initiated at 2 or 6 h post-SCI. Real-time PCR quantification was performed to evaluate anti-inflammatory markers at 1 **(A)**, 4 **(B)**, 7 **(C)**, and 28 **(D)** days after the SCI. Gene expression is normalized to the same-day SCI group. Upregulation of inflammatory gene for medium-dose LPS 0.5 mg/kg treatment groups at 1, 4, 7, and 28 days. *P < 0.05, **P < 0.01, ***P < 0.001 versus SCI (main effect of treatment), *n* = 3, mean + SEM.

Furthermore, the change in anti-inflammatory genes following treatment with a sub-toxic dosage of LPS was assessed. There was a significant treatment main effect for anti-inflammatory gene expression at 1 dpi (*F*_12,73_ = 5.813, *P*< 0.0001), 4 dpi (*F*_12,73_ = 4.577, *P*< 0.0001), and 28 dpi (*F*_12,93_ = 3.88, *P*< 0.0001). There was a trend for upregulated anti-inflammatory gene expression compared to SCI in the medium-dose LPS-2 h group at 1, 4, 7, and 28 dpi (*P*< 0.001 main effects versus SCI). At 28 dpi, there was a significant increase in gene expression with low-dose LPS-2 h treatment groups (*P*< 0.0001, *P*< 0.01 main effect versus SCI for each treatment group, respectively) and driven largely by an increase in IL-10 expression (Figure 4). This effect might be directly associated with the changes in anti- and/or pro-inflammatory factor expression, which provide an inflammatory response environment that favors repair of the structure and function of the injured spinal cord.

#### Administration of medium dose of lipopolysaccharide after spinal cord injury improved functional recovery in rats

**FIGURE 5:**
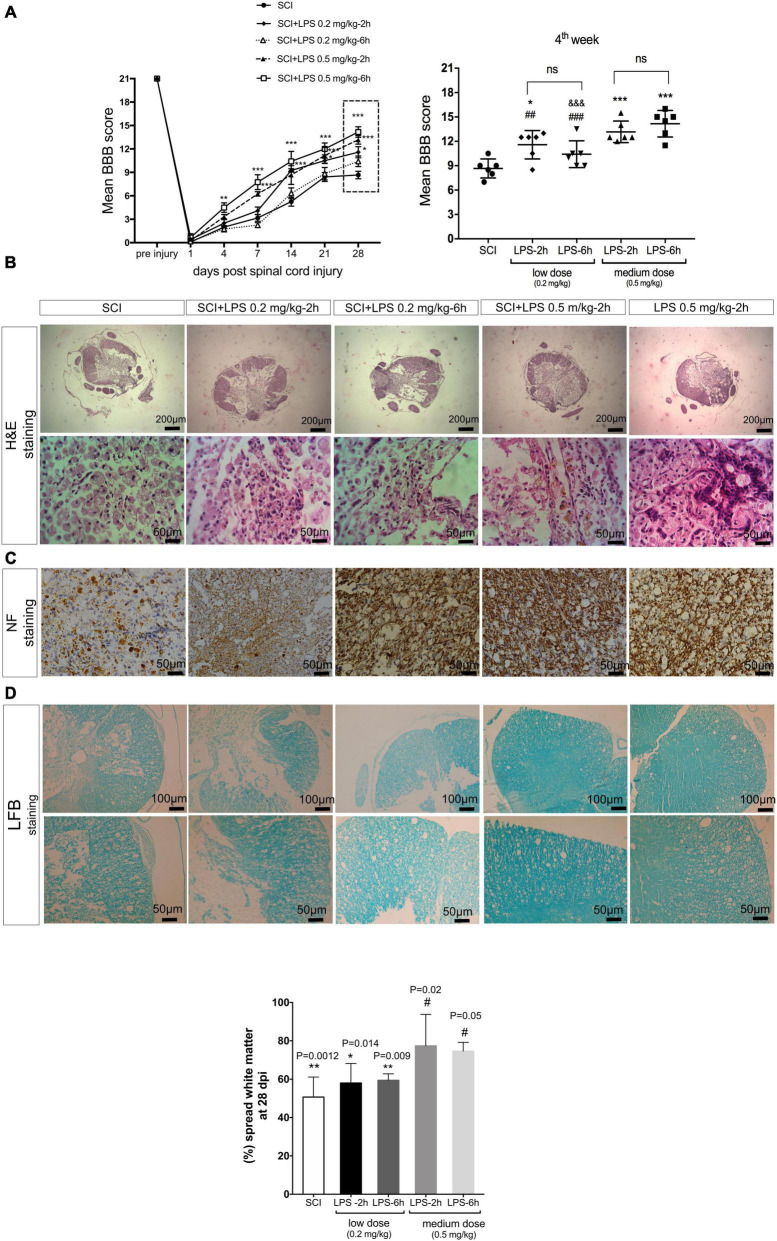
Lipopolysaccharide administration improves hind-limb locomotor function and histological alternations after the SCI. **(A)** Un-treated (SCI) group show spontaneous partial recovery of the hind-limb during 28 days. Animals that received LPS 0.2 mg/kg and LPS 0.5 mg/kg at 2 and 6 h post-treatment showed a gradual increase in BBB score during 28 days post-SCI. Comparison of the average BBB score at 4 weeks after SCI demonstrated that rats received 0.5 mg/kg LPS at 2 or 6 h post-SCI, have higher BBB scores in comparison to another group **(D)**. *n* = 6 for each group. Values represent as means ± SEM. ***P < 0.001 (compared with SCI), ^#^*P* < 0.05, ^##^*P* < 0.01 (compared with LPS 0.5 mg/kg-6 h group at day 28), ^&⁣&⁣&^*P* < 0.001 (compared with LPS 0.5 mg/kg-2 h group at day 28). **(B)** Hematoxylin and eosin stained cross-sections at the injury epicenter. A small number of axonal transections (arrows) were detected. **(C)** NF-200 immunohistochemistry staining illustrated fewer NF-200 positive cells in the SCI group and LPS 0.2 and 0.5 mg/kg increased the NF-200-positive cells at 28 days post-injury in each group. **(D)** White matter area (WMA) at the lesion segment at 4 weeks. WMS represents the spared WMA at the epicenter divided by the WMA of control sections of intact animals. Scale bar, 20 μm. *n* = 3 for each group. *P < 0.05 and **P < 0.01 versus sham. ^#^*P* < 0.05 versus SCI.

Next, hind limb motor function was assessed on day 1, day 4, and then once weekly for the next 4 weeks. Before induction of the SCI, the neurological function of rats in all groups had a normal BBB score (21). About 24 h post-SCI, all the injured rats showed a mean score of zero, which indicates complete hind-limb paralysis. Treatment with medium-dose LPS (0.5 mg/kg) at 2 or 6 h exhibited significant locomotor improvement 1 week after injury compared to the SCI group (*P* < 0.001) after that rapid increases in BBB score continued by 4 weeks. The average score of the LPS-2 h and LPS-6 h groups was 13.1 ± 0.54 and 14.16 ± 0.84, respectively, on day 28 post-SCI. Animals in this range of BBB score (13–16) started a late phase of recovery and showed frequent to consistent plantar stepping and consistent FL-HL coordination. While treatment with low-dose LPS 0.2 mg/kg showed only modest improvement in locomotor function. The average score of LPS-2 h and LPS-6 h was 11.58 ± 0.71 and 10.4 ± 0.67, respectively, showing occasional weight support without FL-HL coordination. The BBB score of the SCI group gradually improved until they reached to plateau at 3–4 weeks, the mean BBB score observed for 4 weeks was at 8.6 ± 0.47, which indicates that the plantar placement with occasional weight support but without forelimb-hind-limb coordination (Figure 5A).

#### Histopathological evaluation

Representative H&E-stained at the lesion center of spinal cord sections from SCI rats or treated with low or medium LPS at 2 and 6 h are shown in Figure 5B. Our result indicated that the SCI group shows great migration of fibroblasts from meninges, and deposition of collagen within the injured lesion results in a fibrous connective tissue formation, which in turn blocks any future axonal regeneration and connection. In the 0.2 mg/kg-2 h group, foamy macrophages (Gitter cells) moderately distributed all around the cavity of liquefaction necrosis, which was generated due to SCI. In 0.2 mg/kg LPS treated 6 h after SCI, besides macrophage infiltration, mild to moderate fibroblast migration and mildly limited collagen deposition were determined. In 0.5 mg/kg dosage of LPS administration at both immediate (2 h) and early (6 h) post-SCI creation, among distributed foamy macrophages, a small number of axonal transection (arrows) was detected. All treatment groups showed better results with regard to hemorrhage compared to the SCI group (Figure 5B).

To determine whether LPS preserves axons after SCI, immunoreactivity with NF-200 as a specific marker of axonal regeneration was performed to detect neurofilament outgrowth at 28 days after injury. Immunohistochemistry of the injured spinal cord stained with NF-200 showed that few NF-200-positive nerve fibers were seen in the SCI group (Figure 5C). There were also a few NF-200-positive cells observed in both low-dose LPS (0.2 mg/kg) treatment groups. In contrast, in medium dose (0.5 mg/kg) LPS-2 and –6 h group’s density of axons in injured tissues was increased and many regenerated NF-200 positive nerve fibers observed in the injured segment. These results indicate that LPS was induced into neurogenesis after SCI. In parallel with the result of the BBB score significant white matter sparing (*P* < 0.05) was observed in both the medium-dose (0.5 mg/kg) LPS-2 and –6 h groups compared to the SCI (Figure 5D).

### Modification of spinal cord microenvironment with lipopolysaccharide and granulocyte colony-stimulating factor treatments prior to bone-marrow mesenchymal stem cell transplantation

#### Characterization of bone-marrow mesenchymal stem cell by flow cytometry

**FIGURE 6:**
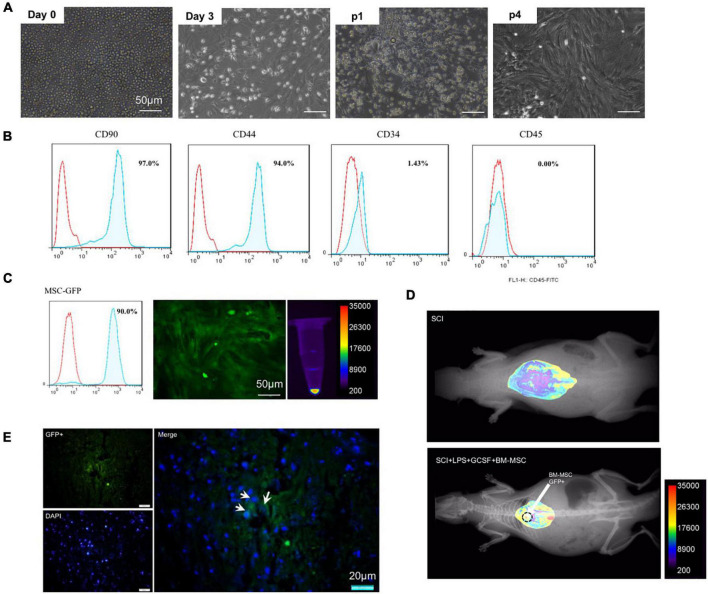
Characterization of BMSC. **(A)** Bright field microscopic of cells at P0–P4 after cultivation. **(B)** Flow cytometry analysis for P4 MSC. **(C)** GFP-positive implanted cell. **(D,E)** Homing of engraftment GFP + MSC detected in the lesion site at 7 dpi.

Bone-marrow mesenchymal stem cell was cultured *in vitro* and exhibited a fibroblast-like morphology (Figure 6A). Flow cytometry showed that undifferentiated cultured BMSCs on the fourth passage expressed immunopositivity for CD44 and CD90, which are bone marrow mesenchymal stem cell markers with each marker being detected in greater than 90% of all cell populations while were negative for the hematopoietic stem cell markers CD34 and CD45 (Figure 6B). Flow cytometry and fluorescent imaging showed the cell’s high GFP-positive (Figure 6C). Fluorescent imaging evaluation revealed that transplanted cells were found in the lesion epicenter of the spinal cord. GFP-positive cells were found in the transplanted group (Figures 6D,E).

#### Combination therapy modulated the inflammatory cytokine level and decrease apoptosis at the lesion site following spinal cord injury

**FIGURE 7:**
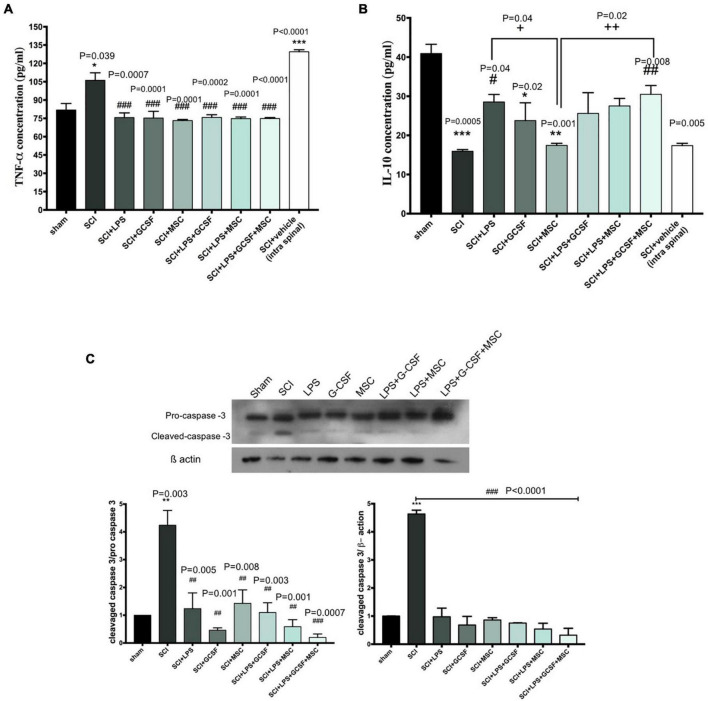
Combination therapy regulates inflammatory cytokine levels and prevents apoptosis in the spinal cord at 7 days post-SCI. **(A)** Levels of TNF-α, **(B)** level of IL-10 in the spinal cord were detected by ELISA on day 7 after the sham operation (*n* = 3/group). ^#^*P* < 0.05, ^##^*P* < 0.01 versus SCI, ^+^*P* < 0.05, ^ + +^*P* < 0.01 versus MSC. **(C)** The protein expression level, determined by Western blot analysis, in the injured spinal epicenter of SCI rat at 7 dpi. Data are presented as mean ± SEM, *P < 0.05, **P < 0.01, and ***P < 0.001 versus sham. # versus SCI or vehicle. One-way analysis of variance (ANOVA) variance followed by Tukey’s post-hoc analysis.

The excessive production and release of inflammatory cytokine following SCI contribute to the secondary injury phase by exacerbating acute damage to spinal cord-resident cells and generating a hostile microenvironment for cell-based therapies. Pro-inflammatory and anti-inflammatory cytokines were altered after SCI. TNF-α and IL-10 as the main regulators of inflammation in the injured CNS. We evaluated the effect of a single-dose LPS treatment or combination of LPS and G-CSF treatment as well as combination therapy with MSC on the level of TNF-α as a pro-inflammatory cytokine and IL-10 as an anti-inflammatory cytokine in the injured spinal cord by examining ELISA at the subacute stage. Analysis showed significantly higher protein levels of TNF-α in both untreated groups (SCI and vehicle) than in the other groups; in contrast, all treatment groups exhibited a significant decrease in the levels of TNF-α. These results suggest that all treatment is capable of reducing the pro-inflammatory cytokine TNF-α for at least at a sub-acute stage in the lesion center (Figure 7A). In contrast, the level of IL-10 in the injured spinal cord shows a significant reduction in the SCI and vehicle group compared to the sham. However, BM-MSC transplanted alone failed to increase IL-10 level. In addition, the protein level of IL-10 increases in all LPS and LPS + GCSF and LPS + GCSF + BM-MSC groups. These results indicate that combined therapy can alleviate the inflammatory reaction following SCI (Figure 7B). The Western blot was performed to determine the apoptotic. SCI, resulted in an increased ratio of cleaved caspase-3/procaspase 3 in the SCI group, significantly lower in all treatment groups at the lesion site of the spinal cord than in the SCI group (Figure 7C).

#### Effects of drug treatment alone or in combination with bone-marrow mesenchymal stem cell, on macrophage polarization

**FIGURE 8:**
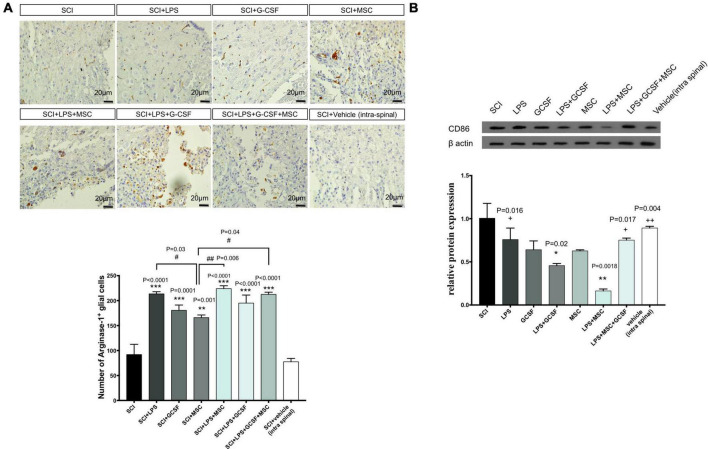
Combination therapy regulates the polarization of macrophage in the lesion center. **(A)** Immunostaining for ARG-1 (as M2 macrophages) performed in the lesion center of spinal cords at 7 dpi from each group of rats (scale bar, 50 μm). **(B)** The protein expression level, determined by Western blot analysis, in the injured spinal epicenter of SCI rats at 7 dpi for each group of rats. Data are presented as the mean ± SEM, *n* = 3 independent experiments. Significant differences between groups are indicated as *P < 0.05, **P < 0.01, and ***P < 0.001 versus SCI. # versus MSC. + versus LPS + MSC. ^#^*P* < 0.05, ^###^*P* < 0.001 versus SCI + MSC, ^+^*P* < 0.05, ^ + +^*P* < 0.01 versus LPS + MSC.

Arg-1 is well known as an anti-inflammatory (M2 macrophage) marker. To further determine the early neuroprotective effect of combinational therapy, we evaluated the effect of combination therapy involved in the anti-inflammatory process induced by Arg-1. Immunohistochemically staining in the lesioned spinal cord tissue was performed 7 days after the contusion SCI. Immunohistochemical staining showed all types of cells expressing Arg-1 including glial cells and neurons. Arg-1-positive cells were mainly located in the gray matter of the lesion center. In the SCI group, the number of Arg-1 expression cells decreased compared to the all treatment group, which represents an inflammatory response (Figure 8A). The Arg-1-positive area increased in all treatment groups compared to the SCI. The number of Arg-1-positive cells in the LPS + GCSF + BM-MSC transplanted group was relatively higher in the other treatment group. Suggesting that modulation of inflammation with combined drugs and after that MSC transplantation promotes Arg-1 as a main anti-inflammatory marker. To determine the pro-inflammatory (M1 macrophage) marker CD86 expression following SCI, CD86 was detected using the western blot analysis. Significantly, higher levels of the CD86 protein were detected following the SCI. In contrast, all treatments suppressed the CD86 protein expression in the injured spinal cord. The decreased CD86 expression indicated that LPS, G-CSF, and BM-MSC can alleviate inflammatory macrophage following SCI (Figure 8B).

#### Combination strategy promotes histopathological change rather than bone-marrow mesenchymal stem cell monotherapy

**FIGURE 9:**
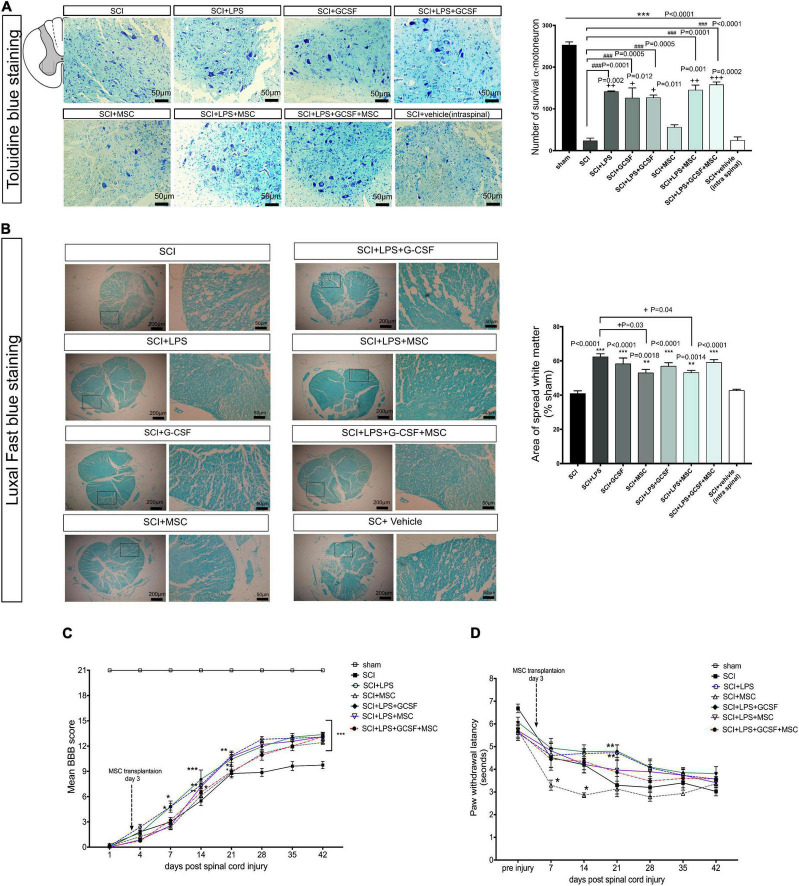
Combination therapy alleviated histophathological change and functional impairment after SCI. **(A)** Toluidine blue staining of the lesion center results in the ventral horn of the spinal cord in the different groups at 7 days after SCI. Drug therapy or combined with MSC transplantation reduces motor neuron loss after SCI. **(B)** Luxol fast blue staining at the injured area. Bar graph showing the percentage of white matter sparing (WMS) compared to sham animals WMS represents the spared WMA at the injured site divided by the WMA of control sections of sham animals. There is no significant difference between treatment groups. **(C)** Motor function was assessed using the Basso, Beattie, Bresnahan (BBB) locomotor rating scale. A gradual recovery of hind-limb locomotion was observed in all rats over the following 42 weeks. Repeated measures two-way ANOVA, Tukey’s post-hoc test. **(D)** Thermal hyperalgesia in the hot plate test was observed during 42 weeks. *P < 0.05, **P < 0.01, and ***P < 0.001 versus SCI. Data represent mean values ± SEM, *P < 0.05, **P < 0.01, ***P < 0.001 versus the SCI group, and ^+^*P* < 0.05, ^++^*P* < 0.01, ^+++^*P* < 0.001 versus the MSC group, *n* = 3 per group. ^###^*P* < 0.001 versus SCI.

In addition to the effect of combination therapy to regulate the inflammatory milieu in the lesion center, we also examined the potential role of combination treatment on structural remodeling including motoneuron survival and white matter tissue sparing at the 7-day post-contusion SCI. Toluidine blue staining of the ventral horn area demonstrated serious lesions, including shrunken neurons and fewer number of survival neurons in the untreated groups (SCI and vehicle groups) as compared with the treatment groups. At the same time, motor neuron survival improved in all treatment groups. In the BM-MSC transplantation alone, the number of motor neurons increased but not significantly compared to SCI or vehicle-treated animals, whereas combination treatment significantly increased the number of motor neurons in the ventral horn compared to the SCI or vehicle. Interestingly, tissue integrity and the number of motor neurons seemed to increase with a combination of the LPS + G-CSF + BM-MSC transplanted group. These results imply that the administration of LPS and G-CSF promotes moto-neuron survival and alleviated pathological loss as well as enhanced the impact of engrafted BM-MSCs in the ventral horn in the injury epicenter (Figure 9A).

Then, we analyzed white matter sparing in Luxol fast blue-stained sections at the lesion site 7 days post-injury. The mean percentage of spared white matter at 1-week post-injury was significantly higher in all treatment groups and had a positive effect on tissue preservation compared to the SCI and vehicle groups. Statistical analysis indicated the BM-MSC and LPS + BM-MSC groups exhibited less white matter area (WMA) at the lesion site compared to the LPS group (*P* < 0.05) (Figure 9B).

To examine whether the microenvironment modified by the combination of therapeutic strategies had any positive effects on histopathological change, we assessed the hyperemia, degeneration, infiltration, and demyelination at the lesion center 7 days after SCI (Table 2). All experimental groups had better results than the SCI and vehicle groups. LPS show significant improvement in infiltration, degeneration, and demyelination. In contrast, in the group that received BM-MSC monotherapy, no significant difference was observed for hyperemia, degeneration, infiltration, and demyelination compare to the SCI and vehicles. When LPS or LPS + G-CSF combined with BM-MSCs all parameters improved compared to the BM-MSC monotherapy. These findings highlight the importance of combining treatment drugs to enhance the BM-MSC effect (Supplementary Table 1).

**TABLE 2:** Histological analysis scores according to the hyperemia, degeneration infiltration, and demyelination between SCI and treatment groups at 1 week post-SCI.

Variable	Group	Mean score (0–3)	SD	Min.	Max.	*P*
Hyperemia	SCI	1.37	0.74	1	3	0.036
	SCI + LPS	0.37	0.44	0	1	
	SCI + MSC	1.87	0.83	0	3	
	SCI + LPS + MSC	0.9	1.3	0	3	
	SCI + LPS + GCSF	0.85	0.69	0	2	
	SCI + GCSF	1	0.92	0	2	
	SCI + LPS + GCSF + MSC	1.25	0.5	0	2	
	SCI + vehicle	1	0.53	0	2	
Degeneration	SCI	2.87	0.35	2	3	<0.0001
	SCI + LPS	1.18	0.59	0	2	
	SCI + MSC	2.68	0.37	2	3	
	SCI + LPS + MSC	1.78	0.69	1	3	
	SCI + GCSF	2.31	0.5	0	3	
	SCI + LPS + GCSF	2.14	0.80	1	3	
	SCI + LPS + GCSF + MSC	1.62	0.44	1	2	
	SCI + vehicle	2.75	0.46	2	3	
Infiltration	SCI	1.87	0.83	1	3	0.02
	SCI + LPS	0.5	0.53	0	1	
	SCI + MSC	2.125	0.99	0	3	
	SCI + LPS + MSC	1.143	0.62	0	3	
	SCI + GCSF	1.75	1.25	0	2	
	SCI + LPS + GCSF	1.28	1.25	0	3	
	SCI + LPS + GCSF + MSC	1	1.02	0	3	
	SCI + vehicle	1.68	0.70	1	3	
Demyelination	SCI	2	0.75	1	3	<0.0001
	SCI + LPS	1	0.5	0	2	
	SCI + MSC	2.18	0.65	1	3	
	SCI + LPS + MSC	1.35	0.47	1	2	
	SCI + GCSF	1.37	0.74	1	3	
	SCI + LPS + GCSF	1.57	0.35	1	2	
	SCI + LPS + GCSF + MSC	0.7	0.63	0	1	
	SCI + vehicle	2.25	0.46	2	3	

Histological score: 0 = (Negative); 1 = (Mild); 2 = (Moderate); 3 = (Severe).

### Combination therapy improves locomotor function and decreases pain hypersensitivity to thermal stimulation

To investigate the beneficial effects of LPS and G-CSF pretreatments on BM-MSC transplantation in a spinal cord contusion injury model, rats of the various treatment groups were assessed by the BBB score (Figure 9C). Neurological function was evaluated between day 1 and 6 weeks after the SCI. The significant motor disturbance in the hind limbs was observed in the SCI group. The average BBB score of the MSC monotherapy group showed relieved neurological damage induced by the SCI, but recovery in motor function started at 21 days post-SCI, with an increase in BBB scores. We observed that in animals that received only a medium dose of LPS or LPS + G-CSF significant increase in motor function started at 7 DPI (*P* < 0.05) and continued until 42 DPI (*P* < 0.001). However, when animals received LPS + BM-MSC or LPS + G-CSF + BM-MSC motor function further increased in BBB score indicating a greater locomotor function. In this combination therapy groups, motor recovery was started at 14 days post-SCI (*P* < 0.01), which indicates that 1 week shows faster recovery than BM-MSC monotherapy, which started at 21 days compared to the SCI group (Supplementary Table 2).

We evaluate the effect of single or combinational therapy on sensory function by analyzing neuropathic pain induced by the SCI. A hot-plate apparatus is used to measure latency in response to a painful thermal stimulus. All treated groups showed an increase in thermal sensitivity compared to the sham control (Figure 9D). A decrease in the response latency following SCI was noted after the 7 days post-injury and sustained until the end of the experiment. But BM-MSC-treated group and SCI group significantly decreased when compared with the pre-injury day (Figure 9D). In contrast, the tendency of LPS and other combined treatment groups is to increase the latency of response but not significantly between groups. However, LPS combining with G-CSF increased the response latency, but only on day 21 when compared with the SCI group, which suggests that either drug treatment or combined use of BM-MSCs could faster alleviate neuropathic pain compared to the BM-MSC alone treatment.

## Discussion

Spinal cord injury is a neurological condition, for which no effective treatments are available to improve recovery from the SCI. Recently, attempts have been made toward restoring the damaged tissue and preventing the spreading of secondary neurodegeneration. Preclinical and clinical studies applied various sources of stem cells as a promising approach for the SCI. Adult stem cells including MSCs have exhibited beneficial effects in the SCI animal model (Shende and Subedi, 2017). A large body of research has shown that transplantation of MSCs after SCI can reduce the cavity size and inflammation and improve functional outcomes (Cofano et al., 2019). However, studies have provided evidence that single-cell therapy failed in clinical trials due to a hostile environment, local neuro-inflammatory pathways, glial scar, and inhibitory factors. Further studies evaluated that combination therapy with drugs or scaffolds either provides more protection for transplanted cells or injured cells; however, less attention is paid to ameliorating the highly inflammatory environment that occurs after SCI.

We run a clinically oriented study to evaluate how turning inflammatory reactions to anti-inflammatory after SCI provides a better outcome for either cell transplantation or other chemical drugs. In the beginning, we did a dose dependency and time dependency to identify the best dose and time for LPS injection. We chose two clinically relevant times at immediate (2 h) and early acute (6 h) post-SCI. Our results are consistent with previous studies indicating the persistence of inflammatory markers over time (4 weeks in this study) and a reduction in the levels of the anti-inflammatory marker at the lesion site (Stirling and Yong, 2008; Harvey et al., 2015; Kwiecien et al., 2020). Thus, this pro-inflammatory mediator might be responsible for the weak spontaneous motor recovery observed in SCI. For this purpose, low-dose LPS was used as an immuonomodulator. It is well known that high-dose LPS (>1 mg/kg) induced sepsis, leading to death (Sardari et al., 2021). However, a sub-toxic dosage of LPS could reduce inflammation in wound infection (Wang et al., 2016), induce microglial activation (Wang et al., 2018), and reduce inflammation after TBI (Eslami et al., 2015). Studies showed that a single injection of sub-toxic dosage LPS (0.1–0.5 mg/kg) 2 h after mild compression SCI could inhibit inflammation 12 h later, which was characterized by a reduction in the number of macrophages recruited to the spinal cord as well as blocked leukocyte recruitment and circulating neutrophils in the injured spinal cord. This result could indicate the protection of the spinal cord from more secondary damage (Davis et al., 2005). Moreover, *in vivo* studies have demonstrated that preconditioning with LPS has a neuroprotective role after SCI via upregulation of Nrf2 expression, which acts as an antioxidant (Li et al., 2016) and promotes M2 marker arginase-1 in microglia cells (Hayakawa et al., 2014). Another experiment revealed that daily LPS administration after dorsal hemi-section in mice increased macrophage number in the spinal cord and accelerated myelin debris clearance by phagocytosis activity (Vallieres et al., 2006). Studies indicated that LPS could not cross the blood-brain barrier (Singh and Jiang, 2004); however, recent studies have shown that peripheral LPS could directly stimulate intact CNS parenchyma by binding their lipoprotein transport (Vargas-Caraveo et al., 2017). Moreover, traumatic CNS injury leads to disruption of the blood-spinal cord barrier and recruitment of inflammatory cells that allow LPS entry into the CNS.

We found that single exposure to the LPS might alter the local immune response in regards to changes in gene expression of immune mediators in all treatment groups. Interestingly, the main effect analysis demonstrates that administration of low–dose LPS (0.2 mg/kg) either 2 or 6 h post-SCI has shown marked induction in the mRNA expression of inflammatory gene up to 4 weeks. Besides these findings, suppression of inflammatory gene expression with LPS 0.5 mg/kg at 1 and 4 dpi was observed. Furthermore, subsequent long-lasting upregulation of anti-inflammatory genes was detected with 0.5 mg/kg-2 h for 28 days. Immediate (2 h post-SCI) administration of LPS 0.5 mg/kg resulted in marked induction in the mRNA expression level of IL-10 in the injured spinal cord. IL-10 is considered as the key anti-inflammatory cytokine, which plays a key role in cross-talk between neurons and glial cells and is also produced by CNS-infiltrating immune cells. It seems that this elevation in the mRNA level of IL-10 directly contributes to TLR4 stimulation by LPS in microglial cells and recruited peripheral macrophage to the injury site (Henry et al., 2009). Our data proposed that LPS (0.5 mg/kg) applied post-SCI reduced the expression of associated pro-inflammatory genes and could alter the inflammatory status toward anti-inflammatory at the gene level. Previous studies did not examine any functional impact of LPS injection on locomotor function.

Our results proposed that there is a dose-dependent effect of LPS on hind-limb motor recovery. It is necessary to receive a single administration of LPS 0.5 mg/kg upon injury to obtain the best therapeutic effect on the reduction of local inflammation and preservation of structural change and greater NF 200 protein after SCI.

In the second part of our study, we investigated whether LPS in combination with G-CSF could enhance the LPS effects and improve outcomes after early MSC transplantation. We applied allogenic cryopreserved MSC, which is more abundant in clinical use. Findings demonstrated controversy about the viability and potential effect of MSC after cryopreservation (Davies et al., 2014). But there is a strong agreement that cryopreservation does not have a negative impact on MSC potential (Davies et al., 2014; Bahsoun et al., 2019). In addition, a recent study revealed that allogenic cryopreserved MSC maintained an immunomodulatory role and neuroprotective role after thawing when applied following SCI in the rat (Rosado et al., 2017).

Previous work with MSC indicates that long-term survival and integration with host tissue did not observe after administration (Torres-Espín et al., 2015), and therapeutic effects may be linked to paracrine activity. Additional evidence indicates that intra-lesional MSC injection has a better survival rate when transplanted 3-days post-SCI (Watanabe et al., 2015). With this evidence, we select 3 days post-SCI to inject intra-lesional MSCs. It was not within the scope of this study to examine the survival rate of MSCs after transplantation and the focus switched beneficial effect on host tissue. Our result showed that the fluorescence did not fade after 4 days post-transplantation.

The neuroprotective effect of G-CSF has been described by many research groups after SCI (Khorasanizadeh et al., 2017). Based on recent studies, G-CSF administered once a day for 5 consecutive days, we examined the concomitant use of G-CSF with LPS following SCI. The intention behind the therapy with G-CSF was to support the neuroprotective effect of LPS treatment. There are data on the combined use of G-CSF with stem cell transplantation in order to improve outcomes after cell transplantation. Our study showed that the administration of G-CSF, for 5 days combined with transplantation of NSC, in addition to their respective therapeutic effects, the combination of G-CSF and NSC might yield synergistic effects (Pang et al., 2019). Studies have investigated the effect of combination therapy with BM-MSCs and GCSF in rats with spinal cord injuries. One study used a simultaneous infusion of adipose tissue-derived MSC and GCSF post-SCI and indicated that a further increase in axonal regeneration was observed (Min et al., 2017). In addition, G-CSF co-treatment with BMSCs in the transection spinal cord model showed a greater motor recovery and reduced apoptotic cells around the lesion center (Luo et al., 2009).

Numerous studies have demonstrated that microglia/macrophage polarization plays an important role in the regulation of inflammatory response, which in turn determines the prognosis of the injury.

Meanwhile, some studies have shown that systemic administration of the drug could induce local polarization of macrophages from a pro-inflammatory phenotype (M1) to an anti-inflammatory phenotype (M2). For instance, Parthenolide, as an anti-inflammatory compound that is derived from medicinal plants, shows a neuroprotective role by inducing a macrophage phenotypic switch to M2 in injured spinal cord mice (Gaojian et al., 2020).

Our results supported that LPS alone and combined with G-CSF provide a permissive environment at the acute phase to promote more effective BM-MSC cell therapy due to the reduction in the expression level of pro-inflammation associated with M1 such as CD86 and TNF-α well as promoting the anti-inflammatory macrophage phenotype *via* high levels of IL-10 and Arg-1 at the injured spinal cord.

The substantial decrease in apoptotic protein caspase-3 is also supported by western blot analysis in tissue sections at the subacute phase, which is a more better outcome in drug therapy compared to the BM-MSC alone. The histological results showed that the transplantation of BM-MSCs after SCI slightly was protected against tissue damage and infiltration and protected neuronal survival in the epicenter of the injury. Moreover, co-treatment with LPS has more beneficial histological changes than MSC alone.

Locomotor recovery analysis showed a relevant benefit of the combination strategy. It seems that this increase in motor neuron number contributed to the achievement of the best functional recovery among the LPS and the combination of LPS with G-CSF and BM-MSC groups, which is consistent with the previous (Sun et al., 2019). Nevertheless, BM-MSC monotherapies did not significantly increase motor neuron survival compared to the drug alone or combination therapy with MSCs after 7 days post-SCI. Therefore, it seems chemical drugs with ease of use may be considered as a potential therapy in the SCI if they correctly pick and use at the proper time. It seems that this increase in motor neuron number and function contributed to the achievement of the best functional recovery among the LPS and combined LPS group with G-CSF and BM-MSC groups, which is consistent with the previous (Sun et al., 2019). In this study, drug therapy alone with LPS or combined with LPS and GCSF led to more rapid recovery, resulting in faster improvement.

## Conclusion

Our study shows using a sub-toxic dose of LPS (0.5 mg/kg), could shift the pro-inflammatory to anti-inflammatory milieu at the lesion site to provide a beneficial micro-environment for MSC transplantation. This study demonstrated that combination therapy provides a better functional outcome following an SCI. These therapeutic effects are associated with the regulation of cytokine production. Furthermore, combination treatment results in motor survival and a decrease in demyelination. Based upon these findings, combination treatment with LPS and G-CSF provides a novel treatment protocol for future clinical trials for enhancing the therapeutic effects of BM-MSC monotherapy in SCIs. Additional studies are needed to determine the detailed mechanisms of the therapeutic effects provided by these combination therapies in SCIs.

## Data availability statement

The original contributions presented in this study are included in the article/Supplementary material, further inquiries can be directed to the corresponding author.

## Ethics statement

All experimental procedures were conducted in accordance with the guidelines for the care and use of laboratory animals observed at the School of Medicine, Tehran University of Medical Sciences (Protocol number: IR.TUMS.VCR.REC. 1396.3157).

## Author contributions

SHa: project administration, writing the original draft, methodology, and analyzed, interpreted, and visualized the data. SHo: perform the experiments. AO and HA: histopathology study. MH: conceptualization, supervision, and writing—review and editing. SE-B, BA, and JA: conceptualization and design the research. All authors read and approved the final manuscript.
