# Supplementary material for: Novel therapeutic approach to slow down the inflammatory cascade in acute/subacute spinal cord injury: Early immune therapy with lipopolysaccharide enhanced neuroprotective effect of combinational therapy of granulocyte colony-stimulating factor and bone-marrow mesenchymal stem cell in spinal cord injury
Source: Front Cell Neurosci. 2022 Nov 23;16:993019. doi: 10.3389/fncel.2022.993019 (PMC9727083; doi:10.3389/fncel.2022.993019)
Supplement: Supplementary file 1 [file Table_1.DOCX]

**Supplementary Information for**

Novel therapeutic approach to slow down the inflammatory cascade in acute /subacute SCI:Early immune therapy with LPS enhanced neuroprotective effect of Combinational therapy of G-CSF, BM-MSC in SCI

Shiva Hashemizadeh^1,2^. Saereh Hosseindoost^3^. Ameneh Omidi^4^. Hossein Aminianfar ^5^. Somayeh Ebrahimi-Barough^6^. Jafar Ai^6^. Babak Arjmand^7,8^. Mahmoudreza Hadjighassem ^1,2^*

1. Department of Neuroscience and Addiction Studies, School of Advanced Technologies in Medicine, Tehran University of Medical Sciences, Tehran, Iran
2. Brain and Spinal Cord Injury Research Center, Neuroscience Institute, Tehran University of Medical Sciences, Tehran, Iran
3. Pain Research Center, Neuroscience Institute, Tehran University of Medical Sciences, Tehran, Iran
4. Department of Anatomical Sciences, Faculty of Medical Sciences, Tarbiat Modares University, Tehran, Iran.
5. Institute of Biomedical Research University of Tehran, Iran
6. Department of Tissue Engineering and Applied Cell Sciences, Faculty of Advanced Technologies in Medicine, Tehran University of Medical Sciences, Tehran, Iran
7. Cell Therapy and Regenerative Medicine Research Center, Endocrinology and Metabolism Molecular- Cellular Sciences Institute, Tehran University of Medical Sciences, Tehran, Iran
8. Metabolomics and Genomics Research Center, Endocrinology and Metabolism Molecular-Cellular Sciences Institute, Tehran University of Medical Sciences, Tehran, Iran.

*Correspondence: Dr Mahmoudreza Hadjighassem

Email: [mhadjighassem@tums.ac.ir](mailto:mhadjighassem@tums.ac.ir)

**Table S1.** Multiple comparisons of histological evaluation scores for hyperemia, degeneration and infiltration and demyelination.

| *Variable* | *Reference Group* | *Comparison Group* | *Score* | *P value* |
| --- | --- | --- | --- | --- |
| *hyperemia* | SCI  (1.37) | SCI+LPS  SCI+MSC  SCI+LPS+MSC  SCI+LPS+GCSF  SCI+GCSF  SCI+LPS+GCSF+MSC  SCI+Vehicle | 0.37  1.87  0.9  0.85  1  1.25  1 | 0.21  0.91  0.95  0.91  0.98  0.98  0.98 |
|  | SCI+LPS  (0.37) | SCI  SCI+MSC  SCI+LPS+MSC  SCI+LPS+GCSF  SCI+GCSF  SCI+LPS+GCSF+MSC  SCI+Vehicle | 1.37  1.87  0.9  0.85  1  1.25  1 | 0.21  0.008  0.87  0.93  0.93  0.62  0.76 |
|  | SCI+MSC  (1.87) | SCI  SCI+LPS  SCI+LPS+MSC  SCI+LPS+GCSF  SCI+GCSF  SCI+LPS+GCSF+MSC  SCI+Vehicle | 1.37  0.37  0.9  0.85  1  1.25  1 | 0.91  0.008  0.31  0.22  0.36  0.37  0.36 |
|  | SCI+LPS+MSC  (0.9) | SCI  SCI+LPS  SCI+MSC  SCI+LPS+GCSF  SCI+GCSF  SCI+LPS+GCSF+MSC  SCI+Vehicle | 1.37  0.37  1.87  0.85  1  1.25  1 | 0.95  0.87  0.31  >0.99  >0.99  >0.99  >0.99 |
|  | SCI+LPS+GCSF  (0.85) | SCI  SCI+LPS  SCI+MSC  SCI+LPS+MSC  SCI+GCSF  SCI+LPS+GCSF+MSC  SCI+Vehicle | 1.37  0.37  1.87  0.9  1  1.25  1 | 0.91  0.93  0.22  >0.99  >0.99  0.99  >0.99 |
|  | SCI+LPS+GCSF+MSC  (1.25) | SCI  SCI+LPS  SCI+MSC  SCI+LPS+MSC  SCI+LPS+GCSF  SCI+GCSF  SCI+Vehicle | 1.37  0.37  1.87  0.9  0.85  1  1 | 0.98  0.62  0.37  >0.99  0.37  0.99  >0.99 |

**Continue Table S1**

| *Variable* | *Reference Group* | *Comparison Group* | *Score* | *P value* |
| --- | --- | --- | --- | --- |
| *degeneration* | SCI  (2.87) | SCI+LPS  SCI+MSC  SCI+LPS+MSC  SCI+LPS+GCSF  SCI+GCSF  SCI+LPS+GCSF+MSC  SCI+Vehicle | 1.18  2.68  1.78  2.14  2.31  1.62  2.75 | <0.0001  0.99  0.0148  0.2526  0.5404  0.0019  0.9999 |
|  | SCI+LPS  (1.18) | SCI  SCI+MSC  SCI+LPS+MSC  SCI+LPS+GCSF  SCI+GCSF  SCI+LPS+GCSF+MSC  SCI+Vehicle | 2.87  2.68  1.78  2.14  2.31  1.62  2.75 | <0.0001  0.0001  0.5058  0.0492  0.0071  0.8055  <0.0001 |
|  | SCI+MSC  (2.68) | SCI  SCI+LPS  SCI+LPS+MSC  SCI+LPS+GCSF  SCI+GCSF  SCI+LPS+GCSF+MSC  SCI+Vehicle | 2.87  1.18  1.78  2.14  2.31  1.62  2.75 | 0.9103  0.0088  0.0764  0.6223  0.9011  0.0134  >0.9999 |
|  | SCI+LPS+MSC  (1.78) | SCI  SCI+LPS  SCI+MSC  SCI+LPS+GCSF  SCI+GCSF  SCI+LPS+GCSF+MSC  SCI+Vehicle | 2.87  1.18  2.68  2.14  2.31  1.62  2.75 | 0.9575  0.8774  0.0764  0.9439  0.6606  0.9994  0.0457 |
|  | SCI+LPS+GCSF  (2.14) | SCI  SCI+LPS  SCI+MSC  SCI+LPS+MSC  SCI+GCSF  SCI+LPS+GCSF+MSC  SCI+Vehicle | 2.87  1.18  2.68  1.78  2.31  1.62  2.75 | 0.2526  0.0492  0.6223  0.9439  0.9992  0.6795  0.6795 |
|  | SCI+LPS+GCSF+MSC  (1.62) | SCI  SCI+LPS  SCI+MSC  SCI+LPS+MSC  SCI+LPS+GCSF  SCI+GCSF  SCI+Vehicle | 2.87  1.18  2.68  1.78  2.14  2.31  2.75 | 0.0019  0.8055  0.0134  0.9994  0.6795  0.2853  0.0071 |

**Continue Table S1**

| *Variable* | *Reference Group* | *Comparison Group* | *Score* | *P value* |
| --- | --- | --- | --- | --- |
| *Infiltration* | SCI  (2) | SCI+LPS  SCI+MSC  SCI+LPS+MSC  SCI+LPS+GCSF  SCI+GCSF  SCI+LPS+GCSF+MSC  SCI+Vehicle | 0.42  2.42  1.14  1.28  1  1.42  1.91 | 0.04  0.98  0.65  0.82  0.46  0.93  >0.9999 |
|  | SCI+LPS  (0.42) | SCI  SCI+MSC  SCI+LPS+MSC  SCI+LPS+GCSF  SCI+GCSF  SCI+LPS+GCSF+MSC  SCI+Vehicle | 2  2.42  1.14  1.28  1  1.42  1.91 | 0.04  0.003  0.82  0.65  0.93   \| 0.46 \| \| --- \| \| 0.08 \| |
|  | SCI+MSC  (2.42) | SCI  SCI+LPS  SCI+LPS+MSC  SCI+LPS+GCSF  SCI+GCSF  SCI+LPS+GCSF+MSC  SCI+Vehicle | 2  0.42  1.14  1.28  1  1.42  1.91 | 0.98  2.42  0.16  0.29  0.08  0.46  0.97 |
|  | SCI+LPS+MSC  (1.14) | SCI  SCI+LPS  SCI+MSC  SCI+LPS+GCSF  SCI+GCSF  SCI+LPS+GCSF+MSC  SCI+Vehicle | 2  0.42  2.42  1.28  1  1.42  1.91 | 0.65  0.82  0.16  >0.99  >0.99  0.99  0.79 |
|  | SCI+LPS+GCSF  (1.28) | SCI  SCI+LPS  SCI+MSC  SCI+LPS+MSC  SCI+GCSF  SCI+LPS+GCSF+MSC  SCI+Vehicle | 2  0.42  2.42  1.14  1  1.42  1.91 | 0.82  0.65  0.29  >0.99  0.99  >0.99  >0.99 |
|  | SCI+LPS+GCSF+MSC  (1.42) | SCI  SCI+LPS  SCI+MSC  SCI+LPS+MSC  SCI+LPS+GCSF  SCI+GCSF  SCI+Vehicle | 2  0.42  2.42  1.14  1.28  1.42  1.91 | 0.93  0.46  0.46  0.99  >0.99  0.98  0.9989 |

**Continue Table S1**

| *Variable* | *Reference Group* | *Comparison Group* | *Score* | *P value* |
| --- | --- | --- | --- | --- |
| *Demyelination* | SCI  (2) | SCI+LPS  SCI+MSC  SCI+LPS+MSC  SCI+LPS+GCSF  SCI+GCSF  SCI+LPS+GCSF+MSC  SCI+Vehicle | 1  2.18  1.35  1.57  1.37  0.7  2.25 | 0.03  0.99  0.47  0.87  0.4650  0.0009  0.99 |
|  | SCI+LPS  (1) | SCI  SCI+MSC  SCI+LPS+MSC  SCI+LPS+GCSF  SCI+GCSF  SCI+LPS+GCSF+MSC  SCI+Vehicle | 2  2.18  1.35  1.57  1.37  0.7  2.25 | 0.03  0.006  0.94  0.62  0.92  0.96  0.003 |
|  | SCI+MSC  (2.18) | SCI  SCI+LPS  SCI+LPS+MSC  SCI+LPS+GCSF  SCI+GCSF  SCI+LPS+GCSF+MSC  SCI+Vehicle | 2  1  1.35  1.57  1.37  0.7  2.25 | 0.99  0.0064  0.17  0.52  0.15  0.0001  >0.99 |
|  | SCI+LPS+MSC  (1.35) | SCI  SCI+LPS  SCI+MSC  SCI+LPS+GCSF  SCI+GCSF  SCI+LPS+GCSF+MSC  SCI+Vehicle | 2  1  2.18  1.57  1.37  0.7  2.25 | 0.47  0.94  0.17  0.99  >0.99  0.38  0.11 |
|  | SCI+LPS+GCSF  (1.57) | SCI  SCI+LPS  SCI+MSC  SCI+LPS+MSC  SCI+GCSF  SCI+LPS+GCSF+MSC  SCI+Vehicle | 2  1  2.18  1.35  1.37  0.7  2.25 | 0.87  0.62  0.52  0.99  0.99  0.09  0.40 |
|  | SCI+LPS+GCSF+MSC  (0.7) | SCI  SCI+LPS  SCI+MSC  SCI+LPS+MSC  SCI+LPS+GCSF  SCI+GCSF  SCI+Vehicle | 2  1  2.18  1.35  1.57  1.37  2.25 | 0.0009  0.96  0.0001  0.38  0.09  0.29  <0.0001 |

Table S2. Comparison of BBB score at different time point in each group after spinal cord injury.

| Group |  |  | Days post SCI |  |  |  |
| --- | --- | --- | --- | --- | --- | --- |
|  | 7 | 14 | 21 | 28 | 35 | 42 |
| **Sham** | 21±0.00 | 21±0.00 | 21±0.00 | 21±0.00 | 21±0.00 | 21±0.0 |
| **SCI** | 3±0.25 | 5.5±0.55 | 8.75±0.52 | 8.875±0.47 | 9.625±0.52 | 9.75±0.42 |
| **SCI+LPS** | 4.8±0.4 ^a^ | 7.37±0.46 ^a^ | 10.88±0.36  ^a^ | 12.81±0.32^a,b^ | 12.94±0.3^a^ | 13.06±0.22 ^a^ |
| **SCI+MSC** | 2.37±0.3 ^b^ | 6.12±0.2 ^b,c^ | 8.87±0.2 ^b,c^ | 11.13±0.15 ^a^ | 12 ±0.1 ^a^ | 12.44±0.13 ^a^ |
| **SCI+LPS+MSC** | 2.56±0.1 | 7.18±0.43 ^a^ | 10.75±0.66 ^a^ | 12.19±0.61 ^a^ | 12.56±0.45 ^a^ | 13.13±0.38 ^a^ |
| **SCI+LPS+G-CSF** | 4.81±0.66 ^a^ | 8.06±1 ^a^ | 10.44±0.79 ^a^ | 11.94±0.59 ^a^ | 13.06±0.44 ^a^ | 13.38±0.27 ^a^ |
| **SCI+LPS+GCSF+MSC** | 3.18±0.33 | 6.5±0.64 | 9±0.59 ^b^ | 10.94±0.6 ^a^ | 12±0.53 ^a^ | 13.25±0.48 ^a^ |

a compared to the SCI

b compared to SCI+ LPS and LPS+G-CSF

c compared to the LPS+MSC

p<0.001.
